# Intact mTOR signaling in gastric X/A-like cells is required for bone homeostasis

**DOI:** 10.3389/fendo.2026.1763507

**Published:** 2026-04-10

**Authors:** Tiange Feng, Wenzhen Yin, Caroline C. Picoli, Linyi Liu, Phuong T. Le, Ziyi Liu, Ruili Yu, May Eltahir, Radfar Zohori, Clifford J. Rosen, Weizhen Zhang, Ziru Li

**Affiliations:** 1 Center for Molecular Medicine, MaineHealth Institute for Research, Scarborough, ME, United States; 2 Clinical Translational Science Center, Beijing Tsinghua Changgung Hospital, Tsinghua University, Beijing, China; 3 University of New England, College of Osteopathic Medicine, Biddeford, ME, United States; 4 Henan Provincial People’s Hospital, People’s Hospital of Zhengzhou University, Zhengzhou, China; 5 Department of Physiology and Pathophysiology, School of Basic Medical Sciences, and Key Laboratory of Molecular Cardiovascular Science, Ministry of Education, Peking University, Beijing, China

**Keywords:** bone, mTOR, stomach, TSC1, X/A-like cell

## Abstract

**Introduction:**

Beyond its primary digestive functions, the stomach serves as an endocrine organ, secreting peptides that regulate appetite and energy balance. Among its enteroendocrine populations, X/A-like cells play a pivotal role in controlling food intake, glucose homeostasis, and lipid deposition. The secretion of X/A-like cell-derived hormones, including ghrelin and nesfatin-1, is regulated by the mechanistic target of rapamycin (mTOR) signaling pathway. However, the role of X/A-like cell mTOR signaling in skeletal metabolism remains unexplored.

**Method:**

Using previously validated and published mouse models with X/A-like cell-specific deletion of *Mtor* or its upstream inhibitor *Tsc1*, we assessed bone phenotypes at 12 and 40 weeks of age under chow-fed conditions. Skeletal effects were also evaluated under pathological bone loss conditions, including estrogen deficiency (ovariectomy) and caloric restriction.

**Results:**

Our findings demonstrate that mTOR signaling deficiency in X/A-like cells compromises bone health in male mice, evidenced by cortical bone loss at 12 weeks and trabecular bone reductions at 40 weeks. Furthermore, X/A-like cell-specific *Mtor* deletion significantly exacerbated bone loss in female mice following ovariectomy, impacting both trabecular and cortical parameters. In contrast, activation of mTOR signaling via *Tsc1* deletion in X/A-like cells did not alter bone mass under either chow *ad libitum* or calorie-restricted conditions.

**Discussion:**

Collectively, these findings identify a previously unrecognized role of gastric X/A-like cell mTOR signaling in the regulation of bone metabolism. Maintenance of intact mTOR signaling in these endocrine cells is necessary for bone homeostasis, revealing a novel gut–bone endocrine axis.

## Introduction

The gastric mucosa serves as the primary interface for the body to sense and absorb nutritional signals, subsequently regulating systemic metabolism. Gastric pathologies and surgical interventions can have significant systemic metabolic consequences that extend to remote organs. For example, bariatric surgeries are highly effective at inducing weight loss but are increasingly recognized for their complex effects on remote organ systems, such as bone loss and increased bone fracture risks (1). The potential mechanisms behind these systemic effects are not fully understood, but emerging evidence points to the crucial role of endocrine factors secreted from the stomach.

Gastric endocrine cells are central to the stomach’s role in metabolic regulation. Among these, X/A-like cells are the second most abundant endocrine population, comprising 20-30% of the total (2). X/A-like cells are the primary source of ghrelin, a key orexigenic hormone that regulates energy homeostasis. Ghrelin’s diverse functions include stimulating appetite by activating orexigenic neurons, promoting lipogenesis, reducing insulin sensitivity, and decreasing energy expenditure (3). Remote tissues can be influenced by X/A-like cell hormones, exemplified by a negative correlation between islet size and plasma ghrelin in ghrelin gene knockout mice and littermate controls (4). Interestingly, a mouse model of X/A-like cell ablation (*Ghrl*-DTR) showed no loss of appetite or body weight, and no resistance to a high-fat diet. However, these mice developed severe hypoglycemia when on prolonged calorie restriction (5), revealing effects that cannot be fully explained by ghrelin’s known functions. This suggests that other factors produced by X/A-like cells also influence systemic metabolism. One such factor is Nesfatin-1, a peptide that acts as a counterpart to ghrelin. Nesfatin-1 suppresses appetite, decreases blood glucose, and improves insulin sensitivity (6). Both ghrelin and nesfatin-1 have been shown to positively affect bone metabolism. Ghrelin stimulates osteoblast proliferation and differentiation *in vitro* (7) and increases bone mineral density *in vivo* (8). Similarly, exogenous nesfatin-1 limits bone loss, preserves bone architecture, and increases bone strength under pathological conditions, such as rheumatoid arthritis and osteopenia (9, 10).

The synthesis and secretion of these X/A-like cell-derived hormones are regulated by the mechanistic target of rapamycin (mTOR) signaling pathway. Our previous study demonstrated that the mTOR signaling pathway inhibits ghrelin production (11) while positively correlates with nesfatin-1 gene (nucleobindin-2; NUCB2) expression in the stomach (12). Specifically, X/A-like cell-specific mTOR gene deletion increases circulating acyl-ghrelin, promotes hepatic fat accumulation, and worsens high-fat diet-induced obesity (13). In contrast, activating mTOR by knocking out its upstream inhibitor, tuberous sclerosis 1 (TSC1), reduced ghrelin production and protected mice from diet-induced obesity and liver fat accumulation (13). However, the musculoskeletal effects of X/A-like cells, especially in the context of mTOR signaling, have not been studied.

In the current study, we utilized these previously validated and published X/A-like cell-specific *Mtor* and *Tsc1* knockout mouse models (13) to investigate skeletal phenotypes, and subjected them into various bone loss conditions, including aging (14), ovariectomy (15), and calorie restriction (16). We found that *Mtor* deletion in X/A-like cells led to bone loss in male mice, but not in females, likely due to the protective effects of estrogen. However, *Tsc1* deletion in X/A-like cells did not alter bone parameters across different ages or under caloric restricted conditions. Collectively, these findings suggest that *mTOR* is required for maintaining bone homeostasis, whereas its activation is not essential for bone accrual.

## Methods

### Animals

All animal procedures were conducted following the Guide for the Care and Use of Laboratory Animals issued by the US National Institutes of Health (NIH Publication No. 8023, revised 1978). The experimental protocols received approval from the Animal Care and Use Committee of the MaineHealth IACUC #2207. Mice were kept in standard plastic rodent cages within a controlled environment (22 °C, with a 12-hour light/12-hour dark cycle, lights on at 7:00 AM). Starting at 4 weeks of age, the mice were assigned to a standard normal chow diet (NCD, TEKLAD GLOBAL 2918; Inotiv). They had free access to a regular chow diet and water unless specified otherwise.

*Ghrl-Cre* mice were donated by Dr. Randy Seeley, and *mTOR^flox/flox^* mice were purchased from Jackson Laboratory (Bar Harbor, ME) (17). Both *Ghrl-Cre* and *mTOR^flox/flox^* lines are of the C57BL/6J strain. Female *mTOR^flox/flox^* mice were bred with male *Ghrl-Cre* mice to generate *Ghrl-Cre*;*mTOR^flox/-^* offspring. Male *Ghrl-Cre*;*mTOR^flox/-^* mice were then intercrossed with female *Ghrl-Cre*;*mTOR^flox/-^* mice to produce *Ghrl-mTOR^-/-^* mice and *Ghrl-mTOR^+/+^* littermates.

*Ghrl-TSC1^-/-^* mice and their *Ghrl-TSC1^+/+^* littermate controls were bred in the same strategy as the *Ghrl-mTOR^-/-^* and *Ghrl-mTOR^+/+^* mice.

### Animal procedures

#### 30% caloric restriction

After acclimating to single-housing and being maintained on a control diet (D17110202i; Research Diets; New Brunswick, NJ) for a week, daily food intake was recorded for another week. Subsequently, mice of 6 weeks of age were transitioned to a 30% caloric restriction diet (D19051601i; Research Diets; New Brunswick, NJ) for 6 weeks, supplied daily at approximately 6 pm, prior to the onset of the dark cycle.

#### Ovariectomy

Female mice at 6 weeks of age underwent ovariectomy. Bilateral ovariectomy was performed by making incisions on both sides of the mice’s dorsal area under isoflurane anesthesia. Then the mice were assigned to *ad libitum* normal chow diet for a duration of 6 weeks.

#### Glucose tolerance test

Mice were fasted overnight (16 hours). Body weight and fasting glucose levels were measured, followed by an intraperitoneal injection of glucose (1 g glucose/kg body weight). Blood glucose was assessed using Bayer Contour test strips at 15, 30, 60, 90, and 120-minute intervals after glucose injection by sampling from the tail tip.

#### Micro-computed tomography analysis

A high-resolution desktop micro-tomographic system (vivaCT 40, Scanco Medical AG, Brüttisellen, Switzerland) was used to assess the trabecular and cortical bone microarchitecture, volume and mineral density in mouse tibiae. Scans were acquired using a 10.5 µm^3^ isotropic voxel size, 70 kVp peak x-ray tube intensity, 114 mA x-ray tube current, 250 ms integration time, and were subjected to Gaussian filtration and segmentation.

Trabecular bone was analyzed for bone volume fraction (Tb. BV/TV, %), trabecular thickness (Tb. Th, mm), trabecular number (Tb. N, mm^-1^), trabecular separation (Tb. Sp, mm), and trabecular bone mineral density (Tb. BMD, mg HA/cm^3^). Cortical bone was analyzed for bone area fraction (Ct. BA/TA, %), cortical thickness (Ct. Th, mm), and cortical tissue mineral density (Ct. TMD, mg HA/cm^3^). All analyses were performed using the Scanco software (Medical AG, version 4.05).

#### Dual-energy X-ray absorptiometry densitometry

Mice were subjected to dual-energy X-ray absorptiometry (DEXA) using a PIXImus Densitometer (GE Lunar Corporation, Fairfield, CT, USA), which was calibrated daily with the metal and plastic phantoms provided by the manufacturer. Mice were placed ventral side down with each limb and tail positioned away from the body. Full-body scans were obtained and X-ray absorptiometry data gathered and processed with manufacturer-supplied software. The head was specifically excluded from all analyses owing to concentrated mineral in skull and teeth. The data were analyzed for the bone mineral content (BMC, mg), bone mineral density (BMD, mg/cm^2^), lean mass (g), and fat mass (g).

### ELISA

Mouse serum C-telopeptide cross-linked type I collagen (CTX-1) and procollagen type 1 N-terminal propeptide (P1NP) concentrations were measured by ELISA kits according to manufacturers’ instructions (Immunodiagnostic Systems).

### Statistical analysis

All mice were randomly assigned to the specified groups. Statistical comparisons were performed using either a Student’s t-test or two-way ANOVA, with Sidak’s multiple comparisons test. The overall effect of genetic knockout on bone parameters was also assessed using two-way ANOVA. All statistical analyses were performed using GraphPad Prism version 9. Graphical data are presented as mean ± SD. A p-value of less than 0.05 was considered statistically significant.

The effect of X/A-like cell-specific mTOR ablation on trabecular bone was evaluated by comparing microarchitectural parameters between the *Ghrl-mTOR^+/+^* and *Ghrl-mTOR^-/-^* groups. To test for an overall difference across all dependent variables simultaneously (e.g., Tb. BV/TV, Tb. N, Tb. BMD), a Multivariate Analysis of Variance (MANOVA) was employed. This approach was chosen to account for the inter-correlation between the bone parameters. If a significant group effect was detected by MANOVA, *post-hoc* analyses were conducted using independent two-sample t-tests to compare individual parameters between genotypes. All p-values from multiple comparisons were adjusted using the Bonferroni correction. For all statistical tests, a p-value of less than 0.05 was considered significant. Statistical analyses were conducted using R statistical software (v4.5.1).

## Results

### mTOR deficiency in gastric X/A-like cells impairs bone health in male mice

**Figure 1:**
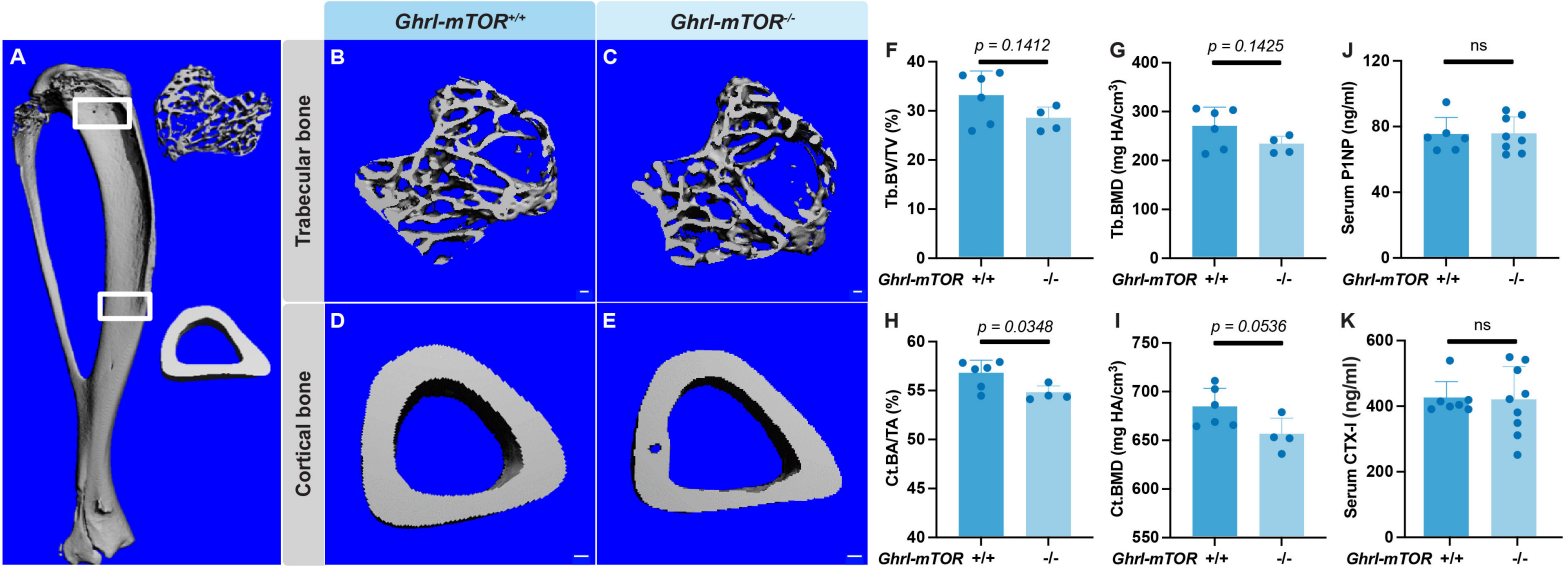
X/A-like cell-specific mTOR deletion causes cortical bone loss in 12-week old male mice. Male *Ghrl-mTOR^-/-^* mice and their *Ghrl-mTOR^+/+^* littermate controls were fed NCD until 12 weeks of age and then euthanized for tissue collection. Trabecular and midshaft cortical bone of the tibiae **(A)** were analyzed by micro-CT. Representative 3D images of trabecular bone **(B, C)** and cortical bone **(D, E)** are shown (scale bar: 100 μm). Trabecular bone volume fraction (Tb. BV/TV) **(F)** and bone mineral density (BMD) **(G)**, as well as cortical bone area fraction (Ct.BA/TA) **(H)** and BMD **(I)**, were measured. Serum levels of P1NP **(J)** and CTX-I **(K)** were measured by ELISA kits. Data is expressed as mean ± SD. Student’s *t-*test.

To investigate the skeletal impact of mTOR signaling in gastric X/A-like cells, male *Ghrl-mTOR^-/-^* mice and their *Ghrl-mTOR^+/+^* littermate controls were maintained *ad libitum* on a normal chow diet (NCD). At 12 weeks of age, no significant change in body weight or random blood glucose levels were observed between genotypes (**Supplementary Figures 1A, B**). Micro-CT analyses were performed to analyze the proximal trabecular and midshaft cortical bone of the tibiae (**Figures 1A–E**). Although the mean values of trabecular bone volume fraction (Tb. BV/TV) and bone mineral density (Tb. BMD) in the *Ghrl-mTOR^-/-^*group were reduced by 14.1% and 13.5%, respectively, compared to the controls, these differences did not reach statistical significance (**Figures 1F, G**). Similarly, trabecular microstructural parameters, including trabecular number (Tb. N), thickness (Tb. Th) and separation (Tb. Sp), were comparable between genotypes (**Supplementary Figures 1C–E**). In the midshaft region, the cortical bone area fraction (Ct. BA/TA) was significantly decreased in the *Ghrl-mTOR^-/-^* mice (**Figure 1H**), and a similar downward trend was observed for cortical bone mineral density (Ct. BMD) (**Figure 1I**). To assess the group effects of X/A-like cell-specific mTOR knockout, an aggregate analysis of all parameters within each bone compartment revealed a significant overall impact (*p* = 0.0198) on the trabecular bone parameters (**Figures 1F, G**: Tb. BV/TV, Tb. BMD, **Supplementary Figures 1C–E**: Tb. N, Tb. Th, Tb. Sp) and a strong trend (*p* = 0.0739) toward a significant impact on the cortical bone parameters (**Figures 1H, I**: Ct. BA/TA, Ct. BMD, **Supplementary Figure 1F**: Ct. Th). To explore whether X/A-like cell-specific mTOR deletion affects the balance between bone formation and resorption, ELISA assays of serum bone turnover markers P1NP and CTX-I were performed (**Figures 1J, K**). However, no intergroup difference was detected.

**Figure 2:**
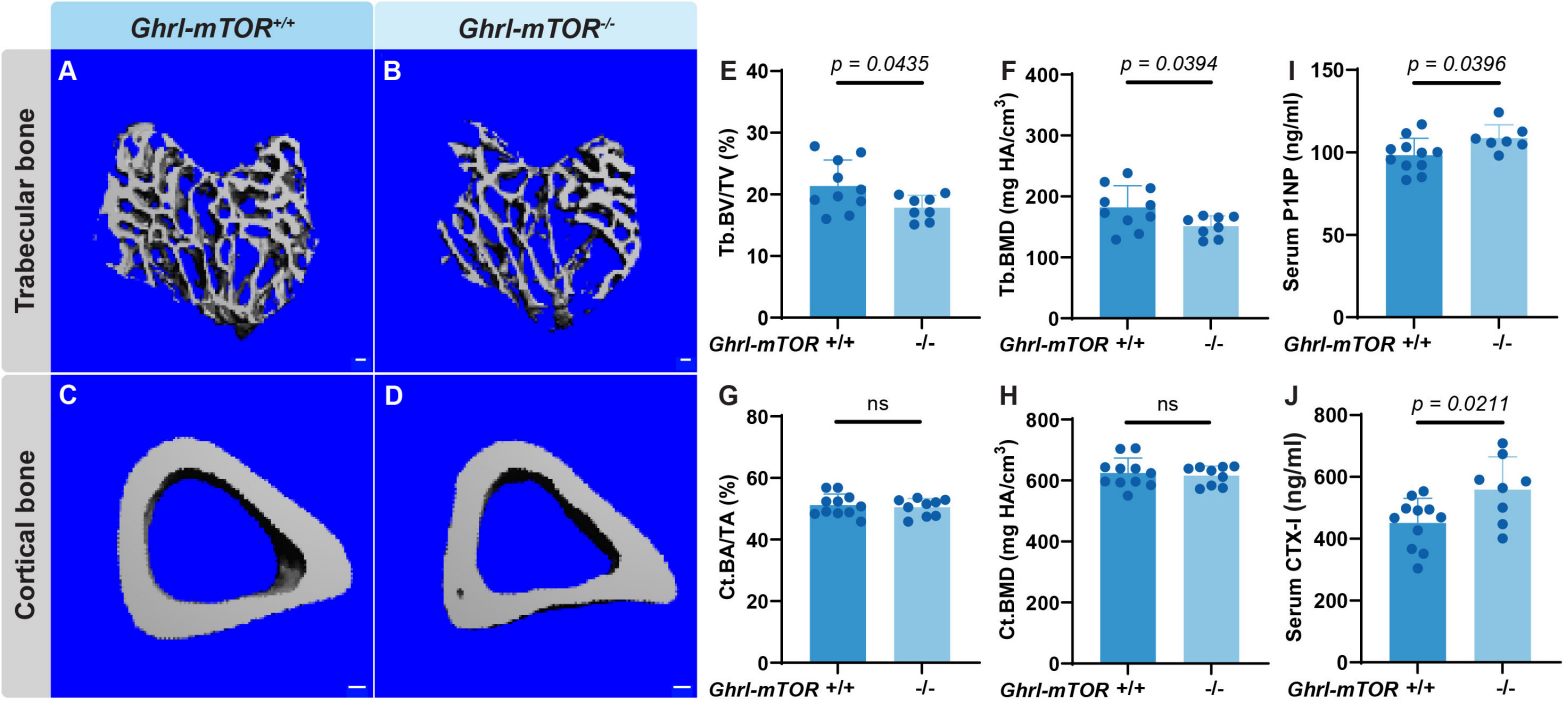
X/A-like cell-specific mTOR deletion reduces trabecular bone in 40-week old male mice. Male *Ghrl-mTOR^-/-^* mice and their *Ghrl-mTOR^+/+^* littermate controls were maintained on NCD until 40 weeks old prior to euthanasia and tissue collection. Trabecular **(A, B)** and midshaft cortical **(C, D)** bone of the tibiae were analyzed by micro-CT and representative images are shown (scale bar: 100 μm). Trabecular BV/TV **(E)** and BMD **(F)**, as well as cortical BA/TA **(G)** and BMD **(H)**, were measured. Serum concentrations of P1NP **(I)** and CTX-I **(J)** were measured using ELISA kits. Data is expressed as mean ± SD. Student’s *t-*test.

The analysis was repeated in 40-week-old NCD-fed male mice. Consistent with the younger cohort, there was no change in body weight or glucose tolerance test compared with their *Ghrl-mTOR^+/+^* littermates (**Supplementary Figures 1G, H**). micro-CT analysis of tibiae (**Figures 2A–D**) revealed a significant reduction in Tb. BV/TV and Tb. BMD in *Ghrl-mTOR^-/-^* mice (**Figures 2E, F**). This was accompanied by mild, non-significant trends toward decreased Tb. N and Tb. Th, and increased Tb. Sp (**Supplementary Figures 1I–K**). No difference in cortical bone parameters was detected between genotypes (**Figures 2G, H**). ELISA assays of circulating bone turnover markers revealed that *Ghrl-mTOR^-/-^* mice had elevated levels of both serum P1NP (10.5%) and CTX-I (23.9%) (**Figures 2I, J**), suggesting a higher bone turnover rate.

Finally, a cross-sectional comparison between the two cohorts confirmed significant age-related bone loss. Bone volume fraction and bone mineral density in both the trabecular and cortical compartments were significantly reduced in 40-week-old mice compared to 12-week-old mice. The magnitude of trabecular bone loss (~30%) was substantially greater than that of cortical bone (~10%), indicating a more dynamic response of trabecular bone to aging.

### X/A-like cell-specific *mTOR* ablation exacerbates ovariectomy-induced bone loss in female mice

Consistent with observations in male mice, X/A-like cell-specific *Mtor* knockout did not affect body weight or blood glucose levels in female mice at 12 or 40 weeks of age (**Supplementary Figures 2A–D**). In these intact females, micro-CT analysis of the tibiae revealed no significant differences between genotypes in any trabecular (Tb. BV/TV, Tb. BMD) or cortical (Ct. BA/TA, Ct. BMD) bone parameters (**Supplementary Figures 2E–L**). A pronounced age-related decline of over 50% in Tb. BV/TV and Tb. BMD was observed in all female mice when comparing 40-week-old animals to 12-week-old counterparts.

Given that the bone loss seen in male *Ghrl-mTOR^-/-^* mice was not present in females under standard conditions, we hypothesized that this discrepancy was due to the protective effects of estrogen. To test this hypothesis, female *Ghrl-mTOR^-/-^* mice and *Ghrl-mTOR^+/+^* littermates underwent ovariectomy (OVX) at 6 weeks of age. In the 6 weeks following surgery, both genotypes exhibited a similar magnitude of body weight gain (**Supplementary Figures 2M, N**). However, the OVX-treated *Ghrl-mTOR^-/-^* mice displayed improved glucose tolerance compared to OVX-treated controls (**Supplementary Figures 2O, P**).

**Figure 3:**
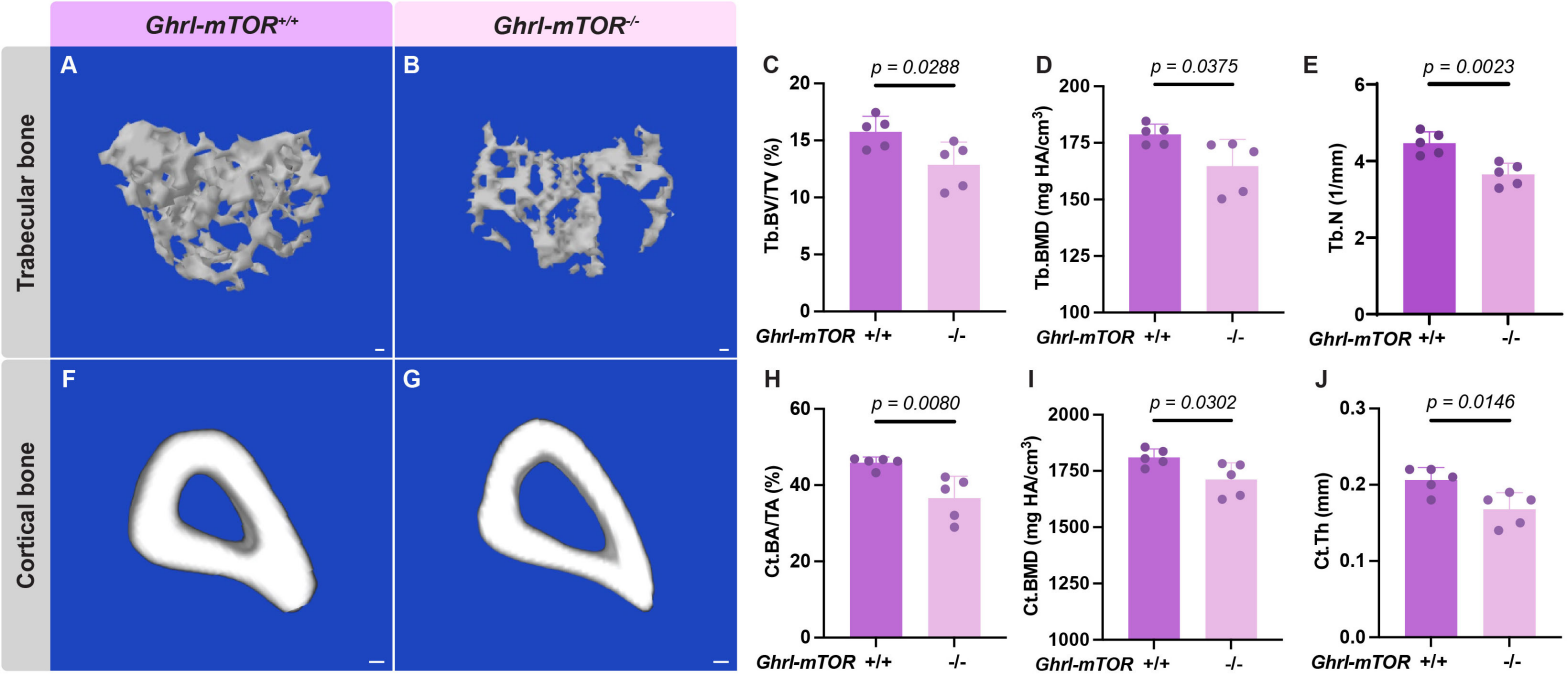
X/A-like cell-specific mTOR deletion exacerbates trabecular and cortical bone deterioration in ovariectomized mice. Female *Ghrl-mTOR^-/-^* mice and their *Ghrl-mTOR^+/+^* littermate controls fed NCD *ad libitum* underwent ovariectomy at 6 weeks of age. They were euthanized at 12 weeks old for tissue collection. Representative micro-CT images of trabecular **(A, B)** and midshaft cortical **(F, G)** bone of the tibiae are shown (scale bar: 100 μm). Trabecular BV/TV, BMD, number (Tb.N) **(C-E)**, as well as cortical BA/TA, BMD, and thickness (Ct.Th) **(H-J)**, were measured. Data is expressed as mean ± SD. Student’s *t-*test.

The OVX procedure unmasked a significant skeletal phenotype, reducing trabecular bone volume for 31.28% (22.92% in intact 12-week old versus 15.75% in age-matched OVX) and cortical bone area fraction for 18.67% (56.42% in intact 12-week old versus 45.89% in OVX). Meanwhile, OVX-treated *Ghrl-mTOR^-/-^* mice exhibited a comprehensive bone loss phenotype compared to their *Ghrl-mTOR^+/+^* littermate controls in both the trabecular (Tb. BV/TV, Tb. BMD and Tb. N) (**Figures 3A–E**) and cortical (Ct. BA/TA, Ct. BMD and Ct. Th) compartments (**Figures 3F–J**).

### *Tsc1* knockout in X/A-like cells does not alter bone mass under normal or calorie-restricted conditions

Results from the *Ghrl-mTOR^-/-^* mice suggest that mTOR signaling in X/A-like cells plays a protective role against bone loss. To further test this, we activated mTOR signaling pathway by deleting its upstream inhibitory tuberous sclerosis complex (TSC) subunit 1 (*Tsc1*; *Ghrl-TSC1^-/-^*).

**Figure 4:**
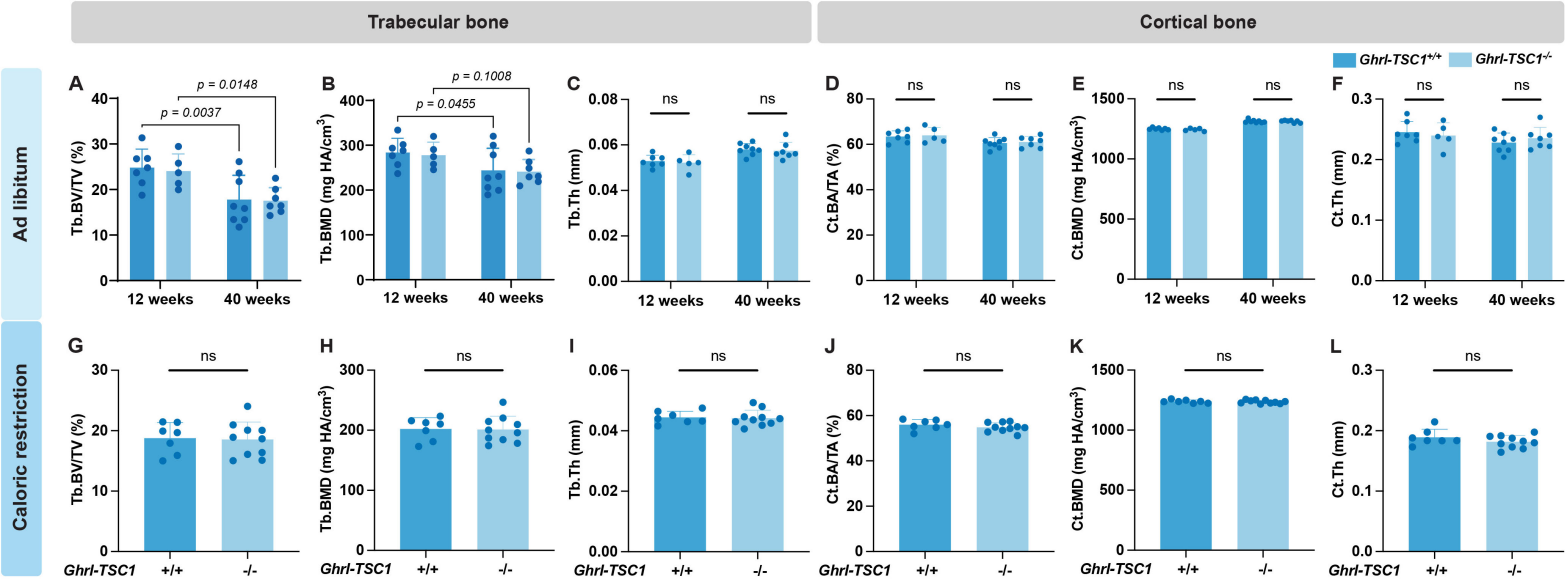
X/A-like cell-specific mTOR activation does not alter bone phenotypes under normal chow diet or caloric restriction. **(A-F)** Male *Ghrl-TSC1^-/-^* mice and their *Ghrl-TSC1^+/+^* littermate controls received NCD *ad libitum* for 12 or 40 weeks, followed by euthanasia and tissue collection. Trabecular **(A-C)** and midshaft cortical **(D-F)** bones of the tibiae were analyzed by micro-CT and bone parameters were quantified. **(G-L)** Male *Ghrl-TSC1^-/-^* mice and their *Ghrl-TSC1^+/+^* littermate controls were subjected to caloric restriction from 6 to 12 weeks of age, followed by euthanasia and tissue collection. Trabecular **(G-I)** and midshaft cortical bones **(J-L)** of the tibiae were analyzed by micro-CT to measure bone parameters as shown. Data is expressed as mean ± SD. Two-way ANOVA for **(A-F)**; Student's t-test for **(G-L)**.

Under a normal chow diet (NCD), male *Ghrl-TSC1^-/-^*mice and their *Ghrl-TSC1^+/+^* littermates were assessed at 12 and 40 weeks of age. Similar with the *Ghrl-mTOR^-/-^* model, no significant inter-genotype differences were found in body weight or glucose tolerance at either age (**Supplementary Figures 3A–C**). Dual-energy X-ray absorptiometry (DEXA) detected no changes in lean or fat mass between genotypes at either age (**Supplementary Figures 3D, E**). The increase in body weight from 12 to 40 weeks was primarily attributed to fat accumulation. No differences were observed between X/A-like cell-specific *TSC1* knockout and control groups in whole-body bone mineral content (**Supplementary Figure 3F**) or bone mineral density (**Supplementary Figure 3G**) either. Micro-CT analyses of the tibiae showed substantial decreases in Tb. BV/TV and Tb. BMD in 40 weeks old mice compared to 12-week old ones (**Figures 4A, B**). However, inter-genotype comparison between *Ghrl-TSC1^-/-^* mice with their *Ghrl-TSC1^+/+^* littermates at both ages revealed no differences in any trabecular or cortical bone parameters (**Figures 4A–F**).

Given the lack of a phenotype under NCD conditions, we next investigated the effects of X/A-like cell-specific mTOR signaling activation under metabolic stress. Caloric restriction (CR) is a well-established intervention known to profoundly impact metabolism and skeletal homeostasis (16, 18). Therefore, we employed a 30% CR model to determine the influences of X/A-like cell mTOR signaling activation on bone health.

*Ghrl-TSC1^-/-^* and *Ghrl-TSC1^+/+^* littermates were placed on the 30% CR diet for 6 weeks, starting at 6 weeks of age. In males, CR induced a predictable decrease in body weight (**Supplementary Figures 4A, F**), primarily driven by fat mass reduction (**Supplementary Figures 4B, C**). While *Ghrl-TSC1^-/-^* males tended (*p* = 0.1725) to lose less weight than the controls, this did not translate to skeletal differences; whole-body BMC and BMD were comparable between genotypes (**Supplementary Figures 4D, E, I, J**). Crucially, micro-CT analyses of the tibiae from these CR-treated males showed no differences in any trabecular or cortical bone parameters (**Figures 4G–L**). In females, CR had a divergent effect, causing an increase in lean mass (more pronounced in *Ghrl-TSC1^-/-^* mice) and only a slight reduction in fat mass (**Supplementary Figures 4K–M, P-R**). Despite these metabolic shifts, female whole-body BMC and BMD were also unaffected by genotype (**Supplementary Figures 4N, O, S, T**).

## Discussion

Our findings reveal a multifaceted role of X/A-like cell mTOR signaling in bone health, with distinct effects observed in male and female mice and across different age groups. Using loss-of-function models, we demonstrate that intact mTOR signaling in X/A-like cells is required for maintaining of bone homeostasis, though this effect appears modest compared to the profound influences of aging or estrogen deficiency. Notably, hyperactivation of mTOR did not increase bone accrual or protect mice from bone loss, suggesting a previously unrecognized, complex, and nonlinear relationship between X/A-like cell mTOR signaling and bone homeostasis.

In the *Ghrl-mTOR^-/-^* model, we consistently observed bone loss in both male and ovariectomized female mice. This indicates a bone-protective effect of the mTOR pathway in X/A-like cells across both sexes. In NCD-fed male mice, 12-week-old *Ghrl-TSC1^-/-^* mice exhibited more pronounced cortical bone loss, while by 40 weeks, trabecular bone loss became significantly more evident. This age-dependent shift in the primary site of bone loss (from cortical to trabecular) aligns with general patterns of bone deterioration, where trabecular bone is often more extensively affected by aging (19, 20). Indeed, the magnitude of changes in micro-CT parameters due to aging substantially exceeded the differences observed between genotypes, indicating that while deficiency in mTOR signaling in X/A-like cells exacerbates bone loss, its overall impact is less pronounced than that of natural aging.

In females, no substantial difference in bone phenotypes was detected between *Ghrl-mTOR^-/-^* and *Ghrl-mTOR^+/+^* mice under normal conditions. Of note, we did not observe sex differences in the extent of mTOR signaling pathway suppression following *Mtor* deletion in X/A-like cells. The skeletal phenotype discrepancies between sexes is likely attributable to the well-documented protective role of estrogen against bone loss. Like humans, where bone loss accelerates significantly in women after menopause due to declining estrogen levels (21, 22), female mice exhibited significantly greater trabecular bone loss between 12 and 40 weeks of age compared to males. The worsened bone loss observed in ovariectomized female mice – where the protective effects of estrogen were removed – further solidifies the indispensable role of estrogen in maintaining bone health and reveals the underlying vulnerability conferred by X/A-like cell-specific *Mtor* deletion.

Our investigation into the *Ghrl-TSC1^-/-^* mice, designed to hyperactivate mTOR signaling, did not show any significant changes in bone phenotypes. This suggests that simply increasing mTOR complex 1 (mTORC1) activity in X/A-like cells does not automatically translate to enhanced bone mass or further mitigation of bone loss. It’s plausible that when *Tsc1* is knocked out, leading to sustained mTORC1 overactivity (23), compensatory mechanisms are triggered to regulate the pathway. Given our previous finding of reduced plasma ghrelin levels in *Ghrl-TSC1^-/-^* mice (24), and a recent meta-analysis suggesting a weak and potentially confounded impact of circulating ghrelin on bone mineral density (25), it’s inferable that the influence of X/A-like cells on bone metabolism is likely not mediated primarily through circulating ghrelin. This implies that other, as yet unidentified, factors or additional intracellular pathways might be at play. These could include the activation of AMPK, an upstream inhibitor of mTOR, in response to energy stress from overactive mTORC1 (26), or the involvement of molecules like Regulated in Development and DNA Damage Response 1 (REDD1), which can suppress mTORC1 activity by releasing TSC2 (27).

We also noted that this study has several limitations. Firstly, while we observed changes in bone parameters, the precise molecular mechanisms by which X/A-like cell mTOR signaling influences bone metabolism remain to be fully elucidated. We measured systemic bone turnover markers (P1NP and CTX-I), but a more detailed investigation into the cellular and molecular pathways linking X/A-like cells to osteoblasts and osteoclasts is needed. This could involve examining direct paracrine signaling from X/A-like cells or identifying specific endocrine factors besides ghrelin that are modulated by X/A-like cell mTOR signaling pathway. Secondly, our study primarily focused on genetically modified mouse models, and while these models provide strong evidence for causality, translating these findings directly to human physiology requires careful consideration. The complexity of human gastric endocrine cells and bone metabolism may involve additional factors not fully recapitulated in mouse models. Thirdly, the lack of a clear bone phenotype in *Ghrl-TSC1^-/-^* mice indicates the possibility of a non-linear relationship between X/A-like cell mTOR activity and bone homeostasis, where an optimal range of mTOR signaling activity exists, and going beyond it doesn’t confer additional benefits, or even elicits counter-regulatory responses. The underlying mechanisms need to be further explored.

Overall, by utilizing X/A-like cell-specific genetic manipulation of mTOR signaling in mouse models, we delineate the causal relationship between this pathway within a specific gastric endocrine cell population and bone health. To our knowledge, this is the first study to specifically investigate the role of mTOR signaling in gastric X/A-like cells in the regulation of bone metabolism, providing novel insights into the complex gut-bone axis that influences skeletal health.

## Data Availability

The original contributions presented in the study are included in the article/**Supplementary Material**, further inquiries can be directed to the corresponding author/s.
