# Supplementary material for: Intact mTOR signaling in gastric X/A-like cells is required for bone homeostasis
Source: Front Endocrinol (Lausanne). 2026 Apr 10;17:1763507. doi: 10.3389/fendo.2026.1763507 (PMC13106587; doi:10.3389/fendo.2026.1763507)
Supplement: Supplementary file 1 [file DataSheet1.pdf]

Supplemental Figures and Legends

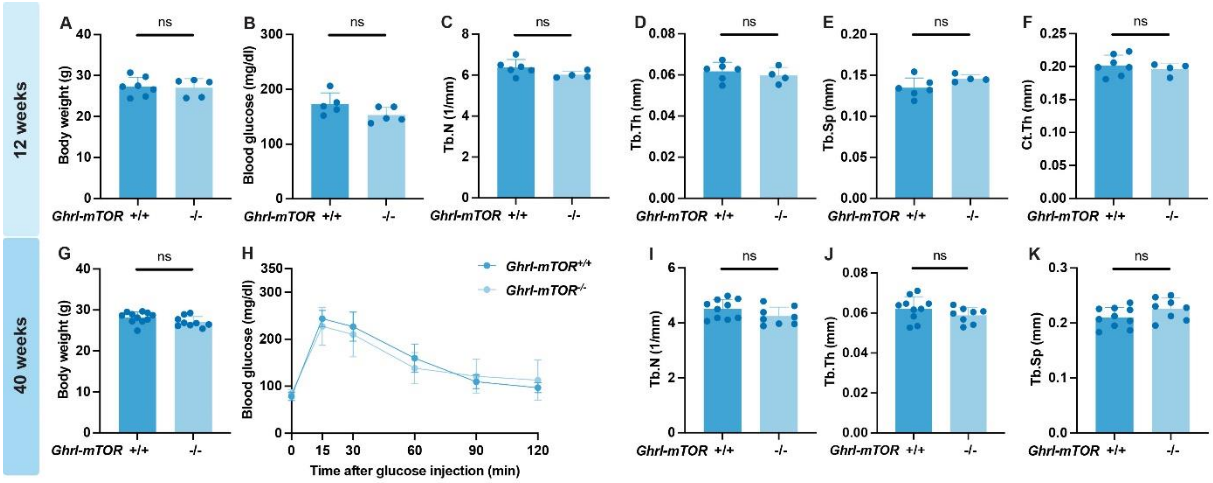

Supplemental Figure 1. Metabolic and bone parameters in male *Ghrl-mTOR*$^{-/-}$ mice at 12 and 40 weeks of age.

A-F. Male *Ghrl-mTOR*$^{-/-}$ mice and their *Ghrl-mTOR*$^{+/+}$ littermate controls were fed a normal chow diet (NCD) *ad libitum* until 12 weeks of age. Body weight (A) and random glucose (B) were measured prior to euthanasia. Tibiae were collected for micro-CT analysis to determine trabecular bone number (Tb. N), thickness (Tb. Th) and separation (Tb. Sp) (C-E), as well as cortical bone thickness (Ct. Th) (F). G-K. Male *Ghrl-mTOR*$^{-/-}$ mice and their *Ghrl-mTOR*$^{+/+}$ littermate controls were maintained on NCD *ad libitum* until 40 weeks of age. Body weight measurement (G) and glucose tolerance test (H) were performed prior to tissue collection. Trabecular bone parameters of the tibiae, including Tb.N, Tb.Th, and Tb.Sp, were determined by micro-CT (I–K). Data is expressed as mean ± SD. Student's *t*-test, except panel H analyzed by two-way ANOVA.

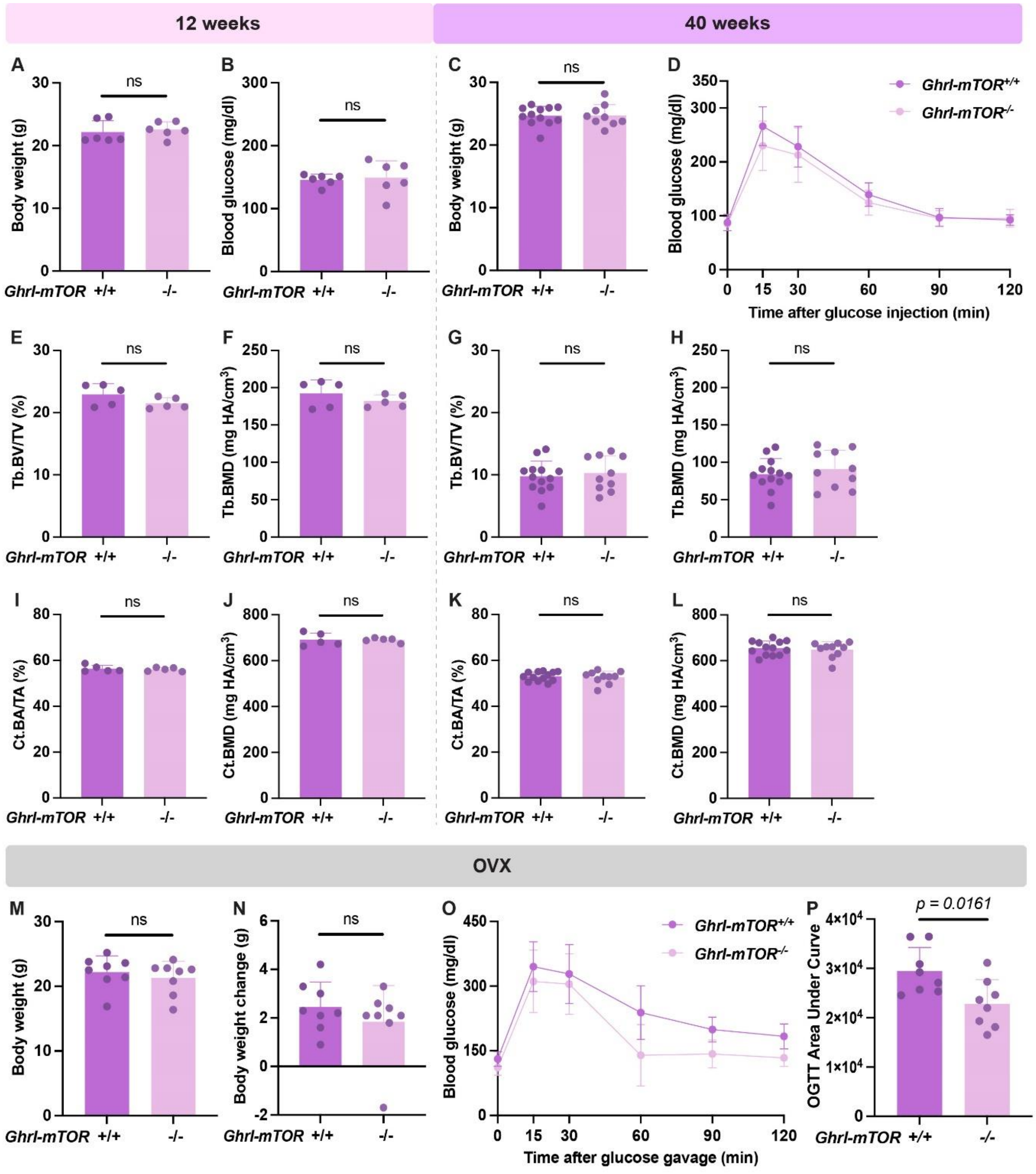

Supplemental Figure 2. No systemic or bone phenotype in intact female *Ghrl-mTOR*$^{-/-}$ mice.

A-L. Female *Ghrl-mTOR*$^{-/-}$ mice and their *Ghrl-mTOR*$^{+/+}$ littermate controls were fed NCD *ad libitum* until 12 weeks of age (A-B; E-F; I-J) or 40 weeks of age (C-D; G-H; K-L), followed by euthanasia and tissue collection. Body weight and blood glucose were measured prior to dissection (A-D). Trabecular BV/TV and BMD (E-H), as well as midshaft cortical BA/TA and BMD (I-L), of the tibiae were analyzed by micro-CT. M-P. Female *Ghrl-mTOR*$^{-/-}$ mice and their *Ghrl-mTOR*$^{+/+}$ littermate controls underwent ovariectomy at 6 weeks of age while maintained on a NCD *ad libitum*. Final body weights and body weight change were determined 6 weeks after

surgery (M-N). Glucose tolerance test was conducted prior to dissection (O-P). Data is expressed as mean ± SD. Student's *t*-test, except panels D and O analyzed by two-way ANOVA.

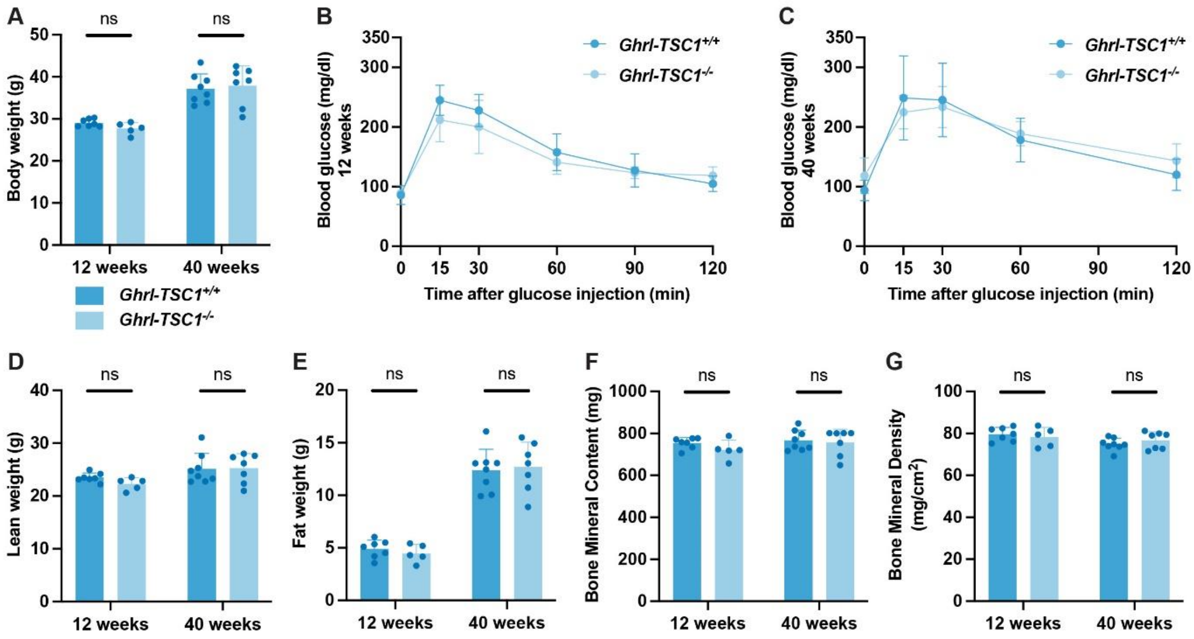

Supplemental Figure 3. No systemic or bone phenotype in male *Ghrl-TSC1*$^{-/-}$ mice at 12 or 40 weeks of age.

Male *Ghrl-TSC1*$^{-/-}$ mice and their *Ghrl-TSC1*$^{+/+}$ littermate controls received NCD *ad libitum* until 12 or 40 weeks of age. Body weight (A), glucose tolerance tests at 12 weeks (B) and 40 weeks (C), and DEXA densitometry (D-G) were conducted prior to tissue collection. Lean mass (D), fat mass (E), whole-body bone mineral content (F) and bone mineral density (G) were measured. Data is expressed as mean ± SD. Two-way ANOVA.

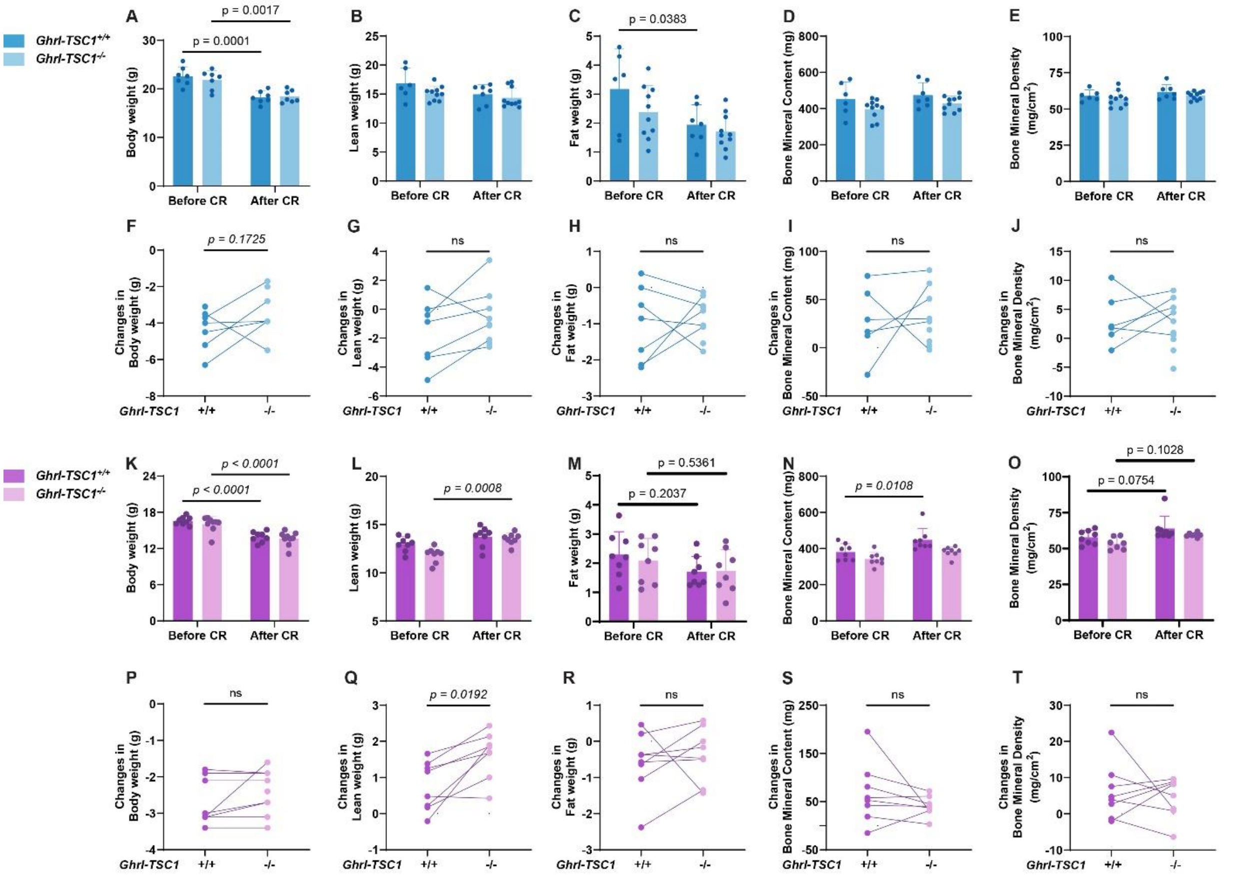

Supplemental Figure 4. X/A-like cell-specific mTOR activation does not alter systemic parameters following caloric restriction, except preservation of lean mass in female mice.

Male (A-J) or female (K-T) *Ghrl-TSC1*$^{-/-}$ mice and their *Ghrl-TSC1*$^{+/+}$ littermate controls were subjected to caloric restriction from 6 to 12 weeks of age. DEXA densitometry was performed prior to caloric restriction and before tissue collection. Body weight (A and K), lean mass (B and L), fat mass (C and M), whole-body bone mineral content (D and N) and bone mineral density (E and O) were measured. Changes before and after caloric restriction were analyzed using paired comparisons (F-J, P-T). Data is expressed as mean ± SD. Two-way ANOVA analysis for panels A-E and K-L; Paired Student's *t*-test for panels F-J and P-T.
